# Clinical outcomes of acute pulmonary embolectomy as the first-line treatment for massive and submassive pulmonary embolism: a single-centre study in China

**DOI:** 10.1186/s13019-020-01364-z

**Published:** 2020-10-21

**Authors:** Wang QiMin, Chen LiangWan, Chen DaoZhong, Qiu HanFan, Huang ZhongYao, Dai XiaoFu, Huang XueShan, Lin Feng, Chen HuaBin

**Affiliations:** 1 Department of Cardiovascular Surgery, Union Hospital of Fujian Medical University, Fuzhou, 350001 Fujian China; 2 Fujian Medical University, Fuzhou, 350001 Fujian China

**Keywords:** Acute pulmonary embolism, Surgical pulmonary embolectomy, Surgical embolectomy, massive pulmonary embolism

## Abstract

**Background:**

Acute pulmonary embolism (PE) is one of the most critical cardiovascular diseases. PE treatment ranges from anticoagulation, and systemic thrombolysis to surgical embolectomy and catheter embolectomy. Surgical pulmonary embolectmy (SPE) indications and outcomes are still controversial. Although there have been more favourable SPE reports over the past decades, SPE has not yet been considered broadly as an initial PE therapy and is still considered as a reserve or rescue treatment for acute massive PE when systemic thrombolysis fails. This study aimed to evaluate the early and midterm outcomes of SPE, which was a first-line therapy for acute central major PE in one Chinese single centre.

**Methods:**

A retrospective review of patients who underwent SPE for acute PE was conducted.Patients with chronic thrombus or who underwent thromboendarterectomy were excluded. SPE risk factors for morbidity and mortality were reviewed, and echocardiographic examination were conducted for follow-up studies to access right ventricular function.

**Results:**

Overall, 41 patients were included; 17 (41.5%) had submassive PE, and 24 (58.5%) had massive PE. Mean cardiopulmonary bypass time was 103.2 ± 48.9 min, and 10 patients (24.4%) underwent procedures without aortic cross-clamping. Ventilatory support time was 78 h (range, 40–336 h), intensive care unit stay was 7 days (range, 3–13 days), and hospital stay was 16 days (range, 12–23 days). Operative mortalities occurred in 3 massive PE patients, and no mortality occurred in submassive PE patients. The overall SPE mortality rate was 7.31% (3/41). If two systemic thrombolysis cases were excluded, SPE mortality was low (2.56%,1/39), evenlthough there were 2 cases of cardiac arrest preoperatively. Patients’ right ventricle function improved postoperatively in follow-ups.There were no deaths related to recurrent PE and chronic pulmonary hypertension in follow-ups, though 3 patients died of cerebral intracranial bleeding, gastric cancer,and brain cancer at 1 year, 3 years, and 8 years postoperatively, respectively.

**Conclusions:**

SPE presented with a low mortality rate when rendered as a first-line treatment in selected massive and submassive acute PE patients. Favorable outcomes of right ventricle function were also observed in the follow-ups. SPE should play the same role as ST in algorithmic acute PE treatment.

## Introduction

Acute pulmonary embolism (PE), is regarded as one of the most critical cardiovascular diseases, which was stratified into high-risk (massive), intermediate risk (submassive), and low-risk PE [1]. PE treatment depends on the stratification, ranging from drug medicine therapy, surgical treatment, interventional therapy and ECMO mechanical support [2]. Surgical pulmonary embolectomy (SPE) [3] was traditionally been used as a final or rescue treatment for acute massive PE when thrombolysis treatment failed to work; therefore, a high SPE mortality rate has been reported. With rapid advanced diagnosis, improved surgical techniques and increased surgeon experience, more favourable SPE results for acute PE have been reported [4]. Increasing excellent SPE reports for acute massive PE extended the indication of SPE for acute PE. SPE has been positively considered as a contemporary algorithm for PE elective treatment, rather than a final rescue tool [2], though there is still controversy regarding SPE mortality rate reports, which vary from 2.3% to 27.2% [5]. Therefore, further research is needed to estimate the role of SPE in algorithmic PE treatment. Most studies were reported by authors from the USA, European countries, and from few developing countries [6]. The aim of this study is to assess the clinical outcomes of SPE for acute PE patients in a single cardiac centre in China.

## Patients and Methods

This was a single-centre, retrospective, uncontrolled study in China. The study protocol was approved by the Medical Ethics Committee of the Union Hospital of Fujian Medical University. Forty-one consecutive acute PE patients who underwent surgical pulmonary embolectomy from July 2005 to August 2019 in our department were included. Informed consent was obtained for every patient. Twenty-four patients underwent emergent surgery, whereas sub-emergent surgery was performed in 17 patients. There were 21 male patients and 20 female patients.

The mean age was 56 ± 11 years (range, 28 -75 years). Acute PE was defined as an acute illness onset with a duration of less than 2 weeks. Patients with acute-on-chronic PE undergoing pulmonary thromboendarterectomy were excluded from this study.

The most common presenting symptoms were dyspnea (*n* = 41, 100%), chest pain (*n* = 13, 31.71%), syncope (*n* = 10, 24.39%), cardiac arrest (*n* = 3, 7.31%), haemoptysis (*n* = 4, 9.75%), and haemodynamic instability (*n* = 24, 58.5%). All patients were examined by echocardiography,and right ventricular dysfunction (RVD) was presented in 34 patients (82.93%).

Computed tomography pulmonary arterial angiography (CTPA) was performed in all patients; however, 2 patients were diagnosed without CTPA because they suffered heart arrest with continued cardiopulmonary resuscitation (CPR) till to surgery. There were 2 cases of thrombosis clot located in the right atrium, and 2 cases showed the clot located in the right ventricle (RV) chamber. There were 18 thrombosis clot cases in the main trunk of the pulmonary artery and 16 thrombus clot cases in the right and/or left pulmonary artery branches.

### Surgical technique

The operation was performed by median sternotomy and cardiopulmonary bypass. To protect the myocardium from ischemia–reperfusion injury, aortic cross-clamping was not used in most patients. During operation, a longitudinal incision was made on the main pulmonary arterial trunk and was extended to the left pulmonary artery. Another incision was made routinely in the right pulmonary artery between superior vena cava and the ascending aorta for further exposure. Thrombus removal was performed en bloc if possible. Gallbladder forceps may be feasible tools to extract thrombotic materials inside distal segmental and sub-segmental pulmonary branches. All branches were carefully inspected. The pulmonary artery tree was irrigated vigorously by a bolus saline flush and aspirated until flesh red blood flowed from the distal pulmonary arterial branch. Extraction should be performed very gently and carefully to avoid damage to the distal smaller pulmonary branches and already infarcted lung tissue. As routine protocol the right atrium and right ventricle were explored and thrombus clots were removed if present. It is important to perform with the beating heart technique to prevent the RV already overloaded from ischemic-reperfusion injury. Postoperatively, a thrombus filter was implanted in the inferior vena cava in the deep venous thrombosis (DVT) patients.

### Post-intervention treatment

The patients received anticoagulation treatments through low molecular weight heparin 6–10 h postoperatively, when the risk of surgical site bleeding disappear.Some patients received a low dose of streptokinase or recombinant tissue plasminogen activator therapy for 3 days when their activated partial thromboplastin time was less than 80 ms. Anticoagulation treatment with heparin was continued until oral warfarin reached the therapeutic range (target international normalised ratio of 2.0 to 3.0). Warfarin was continued for at least one year. All patients were referred to our department for follow-up. Postoperative echocardiography was detected at 2 weeks, 3 months, and 1 year postoperatively.

### Statistical analysis

Statistical analyses were performed using the SPSS software package (SPSS for windows, version 19). Descriptive statistical analysis data are presented as the mean ± standard deviation or median with an interquartile range. The comparison between the data groups was performed using Student’s t-test or the Mann–Whitney U test. *P*-values higher than 0.05 were considered statistically significant.

## Results

There were 24 (58.5%) massive PE cases, and 17 (41.5%) submassive PE cases. All patients were diagnosed with echocardiography, which indicated not only the thrombin clot heart location, but also assessed RV functionality. There were 34 (82.9%) patients who presented with RVD. The RV size was 32.8 ± 9.34 mm, and pulmonary arterial pressure was 73.3 ± 25.9 mmHg. Tricuspid valve regurgitation was 3.6 ± 1.3grades.

In this study, 2 patients accepted systemic thrombolysis as the first choice of treatment, and the treatment failed; the other 39 patients accepted SPE as a first-line therapy. There were 15 patients who were contraindicative of thrombolysis, of whom, 3 patients underwent recent surgery, 4 patients were aged more than 70 years, 4 patients had a right atrium or right ventricle thrombi-clot, 1 patient was pregnant, 1 patient had a brain tumour, 1 patient had an atrial tumour, and 1 patient underwent mitral valve replacement. Another twenty-four PE cases accepted SPE as a first-line treatment on base of the severity of the disease and patients selection.

The concomitant operations were right atrial tumour resectomy in 1 patient and mitral valve replacement in another. CPB time was 103.2 ± 48.9 min. The aortic clamp time was 68.5 ± 29.8 min (10 patients only). Ventilatory support time was 78 h (range, 40–336 h). Intensive care unit stay was 7 days (range, 3–13 days), and hospital stay was 16 days (range, 12–23 days).

Preoperatively, there were 3 patients suffering from cardiac arrest, of whom two received long CPR; one was a 40-min continued CPR, and the other received a 123-min continued CPR until surgery. These 2 patients recovered very well without any brain damage. The other patient, who underwent successful short-duration CPR and accepted systemic thrombolysis as the first choice of therapy but failed, and was resorted to surgical treatment, died of refractory haemorrhage.

There was no death among submassive PE patients, though 3 massive PE patients died postoperatively. One patient, who accepted ST as the first choice of treatment but ST failed to work, and then resorted to SPE, died of uncontrolled massive lung haemorrhage during the operation. One patient died of severe hypoxaemia due to severe ARDS, which led to multiple organ dysfunction even postoperative ECMO support. The other death patient had preoperative successful short time CPR and accepted ST as the first-line treatment but ST failed to work, and resorted to surgery treatment. He died of severe bleeding at the surgical site, which led to multiple organ dysfunction. The overall operative mortality rate was 7.32% (3/41). However, there were 2 patients who accepted thrombolysis as a first-line treatment; if these 2 cases were excluded from the datum, the mortality rate was only 2.56% (1/39).

All patients received inotropic treatment postoperatively and needed prolonged ventilation support, which lasted more than 24 h. One patient needed ECMO support due to severe RVD. Postoperative complications are shown in Table 1. There were 16 patients with DVT; thrombus filters were implanted in their inferior vena cava postoperatively.

**Table 1:** Characteristics of postoperative outcome

Variables	Patients (*n* = 41)
Mean CPB time (minutes)	103.2 ± 48.9 (100%)
Aortic clamp time (minutes)	68.5 ± 29.8 (*n* = 10, 24.39%)
Ventilatory Assist time (hours)	78 h (range, 40–336 h)
ICU Stay (day)	7 days (range, 3–13 days)
Complication (n, %)	
Massive lung hemorrhage	3 (7.32%)
Lung infection	14 (34.1%)
ARDS	4 (9.76%)
Cerebrovascular event	2 (4.87%)
Renal failure	2 (4.87%)
Surgical site bleeding	1 (2.44%)
Multiple organ failure	2 (4.87%)
ECMO support	2 (4.78%)
Diabetes insipidus	1 (2.44%)
Pleura effusion	6 (14.36%)
Mortality rate overall	3 (7.32%, 3/41)
ST Excluded mortality rate	1 (2.56%, 1/39)
Mortality rate for Massive PE	3 (12.5%, 3/24)
Mortality rate for submassive PE	0

*CBP* Cardiopulmonary bypass, *ARDS* Adult respiratory distress syndrome, *ECOM*  Excorporeal oxygerated membrane, *PE* Pulmonary embolism, *ST* Systemic thrombolysis, *ICU* Intensive care union

Follow-ups were conducted in survival patients for an average duration of 2 years (range, 0.5–14 years). The echocardiography datum are shown in Table 2.There was no recurrent acute PE case and chronic pulmonary hypertension.One patient, who had cerebral metastasis cancer, did not wake up and was diagnosed with brain death 2 months after surgical pulmonary embolectomy. One patient died because of cerebral intracranial bleeding 1 year postoperatively. One patient died of gastric cancer 3 years postoperatively. One patient died from brain cancer 8 years postoperatively.

**Table 2:** Preoperative and postoperative echocardiogragh datum of RV

	Pre. *n* = 41	2 weeks Post *n* = 38	3moths Post *n* = 35	1 years Post *n* = 19
Size of RV (mm)	32.8 ± 9.34	25.6 ± 4.32*	24.1 ± 5.08*	23.1 ± 4.61*
Pressure of PA (mmHg)	73.3 ± 25.9	38.6 ± 10.2*	32.9 ± 11.5*	31.2 ± 9.76*
Grade of TR	3.86 ± 1.33	2.62 ± 1.43*	2.13 ± 1.35*	1.21 ± 0.86*

*Pre* Preoperation, *Post* Postoperation, *RV* Right ventricle, *PA* Pulmonary artery, *TR* Tricuspid valve regurgitation

^*^P value<0.05 Compare to “Pre group”

## Discussion

PE symptoms, such as dyspnea, chest pain, haemoptysis, syncope, and arterial hypotension, are often poorly predicted. As the clinical manifestation is low in terms of uniqueness and sensitivity, especially for massive patients with unstable haemodynamics, the therapeutic window is very narrow. It has been reported that about 10% of patients with symptomatic PE die within 1 h of oneset [7]^.^ Therefore, rapid diagnosis is very important. CTPA, which has a sensitivity of 83% and a specificity of 96% [8], is the most common useful tool for rapid diagnosis and risk stratification^.^. CTPA provides a thrombosis clot location visualisation in the main pulmonary arteries and down to at least the segmental level. Acute PE was categorised as massive, submassive, and nor-massive PE. Massive PE [8] was defined as thrombosis clots in the main pulmonary artery as found by CTPA. In our study, 39 patients were diagnosed with CTPA, though 2 patients were diagnosed without CTPA because of continued CPR. If patients have severe haemodynamic instability or are unable to travel for CTPA, transthoracic echocardiography (TTE) should be considered a critical useful tool for diagnosis. TTE provides not only the location of the thrombus but also assesses the structure and function of the RV. In our study, all patients were diagnosed with PE by TTE.

The algorithm treatment for acute PE includes anticoagulation, systemic thrombolysis, catheter intervention and SPE..SPE was considered as a very dangerous treatment for PE due to its high operative mortality of 27.2% to 59% [9]. However, Leacche et al [10] first reported that pulmonary embolectomy mortality was only 6%. The indication of SPE was extended and submassive PE with RVD accepted SPE treatment. The mortality rate of SPE has gradually decreased. Lehnert [11] reported SPE mortality rate of 6%, and more excellent results of SPE were reported [12–14]. Cho [15] reported that SPE had a lower cardiac mortality risk than thrombolysis in PE patients with haemodynamic stability. Increasingly good SPE results indicated that SPE should not only be a rescue therapy for patients when ST failed, but it should also be considered as the first-line PE treatment choice. However, there is still controversy regarding SPE outcomes. Park [16] reported a 14.8% surgical treatment mortality rate. Reza [6] recently reported 36 massive PE surgical treatments with 10 deaths and a mortality rate of 27.8%.

In our centre, there were 41 PE patients who underwent surgical embolectomy over 14 years, of whom 3 patients died postoperatively. The overall operative SE mortality rate was 7.32%. There were 39 PE patients who received SE as first-line treatment, of whom 1 patient died, and the mortality rate was 2.56% (1/39). The causes of death were operative haemorrhage in 2 patients and severe hypoxia in 1 patient. It was coincidental that both massive PE deaths were in patients who accepted ST as their first line of treatment but ST failed to work and then resorted SPE. ST seemed to increase the risk of operative haemorrhage and mortality of SPE. This finding was verified by the study of Aymard [15], who found that SPE had a lower early mortality rate (SPE: 3.6% versus ST 13.5%). whereas early mortality was 27% in those patients treated initially with thrombolysis and subsequently requiring SPE.. For massive PE patients with severe unstable haemodynamics or cardiac shock, if ECMO is unavailable and once ST treatment fails, patients would be faced with deteriorated haemodynamics and a high operative haemorrhage risk. In such case, SPE is a rescue treatment, and a higher SPE morbidity and mortality may occur. However,,SPE is the only choice of treatment when ST fails, if ECMO unvalaible, SPE should be carried out as rapidly as possible for sake of rescue life. A recent study [17] showed that patients treated with systemic thromnolysis have a higher cardiac mortality risk than SPE. Most PE patients without haemodynamic instability, who are afraid of surgical trauma and potential injury from cardiopulmonary bypass, are more likely to accept ST as a first-line treatment when there are no ST contraindications. Considering the favourable early and long-term outcomes in our study with no cases of chronic thromboembolic pulmonary hypertension, it is reasonable to consider SPE as an alternative therapy for submassive PE.

The most common risk factor for surgical treatment is preoperative cardiac arrest [18], which causes a mortality rate of up to 59%. Keeling [19] reported that the in-hospital mortality of patients with preoperative CPR was significantly higher (9/28,32.8%) than in those without CPR (16/186, 8.6%). Takahashi [20] reported that 73% of patients who received CPR for longer than 30 min died after pulmonary embolectomy. Recent reports showed that combining ECMO with SPE improved the outcome of massive PE surgery significantly, especially in massive PE with CPR [21]^.^ However, in developing countries, ECMO is not available in every cardiac centre. In this situation, further treatment for PE with CPR is very challenged. There were 3 patients who had preoperative cardiac arrest in this study, of whom 2 underwent SE as a first-line treatment even after continued CPR, which lasted more than 40 min, and achieved very good recovery without any brain damage. However, the other patient with successful 3 min of CPR accepted thrombolysis as first line treatment but failed, and resorted to SPE. This patient died of refractory surgical site bleeding. In the light of our experience, SPE seemed to be more effective in treatment PE with cardiac arrest when a surgical expertise team was available. SPE as an initial therapy can avoid refractory operative haemorrhage due to thrombolysis. In this study, 2 patients with long-duration CPRs achieved very good SPE recovery without any brain damage. The decisive factor was that cardiac arrest was witnessed by us on-site, and rapid effective CPR started on time and continued till to surgery. In fact, during the study period, there were several PE cases with successful CPR, who were transferred from another hospital to our centre; however, the pupillary light reflex disappeared and the pupils were dilated and fixed, indicating cerebral death. The patients were declined SPE because there was no possibility of cerebral resuscitation. For PE with long-duration CPR, further treatment selection depends on the assessment of cerebral resuscitation rather than CPR duration.

ECMO has been recommended as the standard of management in PE treatment centres [22]. Acute massive PE with pre-operative significant haemodynamic instability with or without the need of CPR requires ECMO as an advanced life support. Postoperative sever hypoxemia and severe RVD may require ECMO support. In this study, 2 patients required ECMO support postoperatively. One patient had postoperative severe RVD who survived after 3 days of ECMO support. The other patient had severe hypoxaemia and died of multiple organ failure even after 1 week of ECMO support. Use of ECMO is associated with a high complication and the place of ECMO in the algorithm of PE treatment need to be further investigated [23].

Surgical techniques have improved over the past decades, and surgery under cardiopulmonary bypass with the”beating heart” technique is recommended as a standard protocol in new guidelines [2]. Aortic cross-clamping causes ischemia–reperfusion myocardium injury and deteriorates RVD. In this study, there were 31 SPE surgeries performed without aortic cross-clamping. This may benefit RV function, and may be a reason for the low rate of postoperative ECMO support.

Massive lung haemorrhage was an SPE-related complication. There were 3 cases of massive lung haemorrhage postoperatively in the study. One patient died during the operation because of an uncontrolled large amount of bleeding in the lung. Two other patients survived. Massive lung haemorrhage is often due to injured pulmonary arterial vasculature. During surgery, in case of pulmonary massive haemorrhage, it is necessary to open the pleural cavity and locate the responsible pulmonary artery and repair it with a 6–0 polypropylene suture. In order to avoid damage to the infarcted smaller pulmonary artery, when removing the thrombus clot in the distal pulmonary arterial branches, it is forbidden to perform extraction without visualisation. It is difficult to extract smaller clots deep in the distal pulmonary arterial branches. We used heparin saline to vigorously irrigate the pulmonary arterial branches and aspirated them until flesh red blood flowed from the distal pulmonary arterial branch. The alternative option [11] was retrograde pulmonary venous perfusion with haparin saline, which was useful to remove a very small clot in the distal pulmonary arterial branches.

## Conclusion

In this study, with improvement of surgical technique and selection of patients, SPE was a safety and effective treatment for acute PE. There were no mortality of submassive PE patient and 3 mortalities of massive PE Patient. When the first-choice treatment was SPE, the overall mortality was low 2.56% (1/39), even though there were 2 cases of long duration preoperative CPR, which indicated the risk of SPE was low. If 2cases with ST as first treatment were included, the overall mortality rate of SPE was 7.32%(3/41).Systemic thrombolysis increased SPE mortality due to haemorrhage in this study. In respect of safety, SPE can play the same role as ST in the treatment algorithm for acute PE. Echocardiography showed right ventricular function improved in the early and midterm follow-up. However, the study was retrospective, and the sample size was rather small; further studies are needed to assess the role of SPE in the treatment algorithm for acute PE.

## Data Availability

Please contact author for data requests.

## Abbreviations

PE: Pulmonary embolism
SPE: Surgical pulmonary embolectomy
ST: Systemic thrombolysis
RVD: Right ventricular dysfunction
CTPA: Computed tomography pulmonary arterial angiography
CPR: Cardiopulmonary resuscitation
CPB: Cardiopulmonary bypass
DVT: Deep venous thrombosis
TTE: Transthoracic echocardiography
ARDS: Adult respiratory distress syndrome
ECMO: Extracorporeal membrane oxygenation
